# Resveratrol Produces Neurotrophic Effects on Cultured Dopaminergic Neurons through Prompting Astroglial BDNF and GDNF Release

**DOI:** 10.1155/2012/937605

**Published:** 2012-11-28

**Authors:** Feng Zhang, Yan-Ying Wang, Hang Liu, Yuan-Fu Lu, Qin Wu, Jie Liu, Jing-Shan Shi

**Affiliations:** ^1^Department of Pharmacology and Key Lab of Basic Pharmacology of Guizhou, Zunyi Medical College, Zunyi 563099, China; ^2^Pharmacy School, Zunyi Meidcal College, Zunyi 563099, China; ^3^University of Kansas Medical Center, Kansas City, KS 66160, USA

## Abstract

Increasing evidence indicated astroglia-derived neurotrophic factors generation might hold a promising therapy for Parkinson's disease (PD). Resveratrol, naturally present in red wine and grapes with potential benefit for health, is well known to possess a number of pharmacological activities. Besides the antineuroinflammatory properties, we hypothesized the neuroprotective potency of resveratrol is partially due to its additional neurotrophic effects. Here, primary rat midbrain neuron-glia cultures were applied to investigate the neurotrophic effects mediated by resveratrol on dopamine (DA) neurons and further explore the role of neurotrophic factors in its actions. Results showed resveratrol produced neurotrophic effects on cultured DA neurons. Additionally, astroglia-derived neurotrophic factors release was responsible for resveratrol-mediated neurotrophic properties as evidenced by the following observations: (1) resveratrol failed to exert neurotrophic effects on DA neurons in the cultures without astroglia; (2) the astroglia-conditioned medium prepared from astroglia-enriched cultures treated with resveratrol produced neurotrophic effects in neuron-enriched cultures; (3) resveratrol increased neurotrophic factors release in the concentration- and time-dependent manners; (4) resveratrol-mediated neurotrophic effects were suppressed by blocking the action of the neurotrophic factors. Together, resveratrol could produce neurotrophic effects on DA neurons through prompting neurotrophic factors release, and these effects might open new alternative avenues for neurotrophic factor-based therapy targeting PD.

## 1. Introduction

Parkinson's disease (PD) is one of the most common neurodegenerative diseases characterized by a selective dopamine (DA) neuronal loss in the substantia nigra (SN) of the ventral midbrain. Although current treatments are mainly used for symptomatic controls, their long-term application is associated with complications and could not halt the neurodegeneration [1]. A growing evidence showed that glia, particularly astroglia and microglia, played an important role in neurodegenerative disorders and had become the prime targets for therapy [2–4]. Astroglia are known to serve a number of housekeeping functions, such as the maintenance of the extracellular environment and the stabilization of neuron and glial cells communications in the brain [5]. Most importantly, astroglia are the major source of various neurotrophic factors, such as brain-derived neurotrophic factor (BDNF) and glial cell line-derived neurotrophic factor (GDNF) [6]. It has become increasingly evident that neurotrophic factors are indispensable for the maintenance and neuronal protection in the developing and adult brain [7]. Furthermore, lack of neurotrophic factors resulted in the neuronal loss and the progression of neurodegenerative diseases [8, 9]. Thus, astroglial neurotrophic factors generation might hold a promising therapeutic potential for the treatment of neurodegenerative diseases.

Resveratrol (3, 4′, 5-trihydroxy-trans-stilbene), a natural nonflavonoid polyphenol, is naturally present in red wine and grapes with a widely potential benefit for health [10]. It was well known to possess a great number of pharmacological activities such as antioxidant, cardioprotective, anti-inflammatory, and anticancer properties [11]. In addition to these beneficial actions, growing interest has been focused on its neuroprotective effects on ischemia, seizure, and neurodegenerative diseases [12]. Moreover, resveratrol was found to improve hippocampal atrophy via enhancing neurogenesis and suppressing the apoptosis of granular cells in chronic fatigue mice [13]. 

Our previous studies indicated that resveratrol produced neuroprotection against lipopolysaccharide- (LPS-) induced DA neurodegeneration through its anti-inflammatory activities and increased neurotrophic factors release in primary astroglia cultures [14, 15]. Besides the antineuroinflammatory properties, we attributed the potential neuroprotective potency of resveratrol to its additional neurotrophic effects derived from astroglia. Here, primary rat midbrain neuron-glia cultures were applied to investigate the neurotrophic effects mediated by resveratrol on DA neurons and further explore the role of neurotrophic factors in its actions. 

## 2. Materials and Methods

### 2.1. Animals and Materials

Female Wistar rats (200–300 g) were purchased from the Experimental Animal Centre of the Third Military Medical University (Chongqing, China; Specific-Pathogen-Free Grade II; Certificate no. scxk 2002003). Housing and breeding of the animals were performed in strict accordance with Animal Care and Use Guidelines in China. 

Resveratrol and the polyclonal antityrosine hydroxylase (TH) antibody (1 : 1000) were bought from Sigma Chemical Co. (St. Louis, MO, USA). The polyclonal anti-BDNF (1 : 1000) and anti-GDNF (1 : 1000) antibodies were obtained from Abcam (Cambridge, MA, USA). The Vectastain avidin-biotin complex (ABC) kit was purchased from Vector Laboratories (Burlingame, CA, USA). Enzyme-linked immunosorbent assay (ELISA) kits were obtained from Promega (Madison, WI, USA). All the cell culture materials were purchased from Invitrogen (Carlsbad, CA, USA). 

### 2.2. Primary Rat Midbrain Neuron-Glia and Neuron-Astroglia Cultures

Primary neuron-glia cultures were prepared from the ventral midbrain tissues of embryonic day 14-15 rats. The whole brain was aseptically removed, and the mesencephalon was dissected. After the blood vessels and meninges were removed, the mesencephalic tissues were dissociated by the mechanical trituration, and the dissociated cells were seeded at 1 × 10^6^/mL in poly-D-lysine-coated 24-well plate. Seven-day-old cell cultures were performed for drug treatments. At the time of treatment, cultures were composed of 10% microglia, 50% astrocytes, 40% neurons, and 1% TH-immunopositive neurons [16]. Primary neuron-astroglia cultures were prepared from inhibiting microglial proliferation with 1.5 mM leu-leu methyl ester (LME) added to primary neuron-glia cultures 24 h after seeding the cells. Seven days after initial seeding the cultures were used for drug treatment, and the percentage of microglia in the cultures was <1% [17].

### 2.3. Primary Rat Microglia- and Astroglia-Enriched Cultures

Primary microglia- and astroglia-enriched cultures were obtained from the whole brains of 1-day-old rat pups [17]. After a confluent monolayer of glial cells was established, microglia were separated from astroglia through shaking the flasks, and primary microglia-enriched cultures were 95–98% pure for microglia. The remaining astroglia were detached with trypsin-EDTA and seeded in the culture medium. After two or three consecutive passages, immunocytochemical analysis indicated primary astroglia-enriched cultures consisted of >98% astroglia. 

### 2.4. Primary Rat Neuron-Enriched and Neuron-Microglia Cultures

Primary rat midbrain neuron-enriched cultures were prepared from suppressing glial proliferation with 6–8 *μ*M cytosine *β*-D-arabinofuranoside (ara-c) added to primary neuron-glia cultures 24 h after seeding the cells [17]. Seven days later the neuron-enriched cultures consisted of 90% neurons, 10% astroglia, and <0.1% microglia. Primary neuron-microglia cultures were obtained by adding 10% (5 × 10^4^/well) primary microglia from primary microglia-enriched cultures back to the neuron-enriched cultures [16].

### 2.5. [^3^H] DA Uptake Assay

Primary neuron-glia cultures were incubated at 37°C with [^3^H] DA in Krebs-Ringer buffer for 20 min. Liquid scintillation counting was performed to detect the radioactivity. Mazindol was used to block the nonspecific uptake for DA uptake. After the cultures were washed for three times with ice-cold Krebs–Ringer buffer and lysed with NaOH, the lysate was mixed with scintillation fluid, and radioactivity was measured through the liquid scintillation counter. The specific [^3^H] DA uptake was calculated by subtracting the radioactivity amount obtained in the presence of mazindol from that obtained in the absence of mazindol [18].

### 2.6. Immunocytochemical Staining

Dopaminergic neurons were recognized with an anti-TH antibody. Primary neuron-glia cultures were fixed by formaldehyde and treated with 1% hydrogen peroxide followed by sequential incubation with blocking solution. Then, the cultures were incubated with primary anti-TH antibody overnight at 4°C and biotinylated secondary antibody at room temperature for 2 h. Consequently, the cultures were incubated with Vectastain ABC reagents for 40 min, and the color was developed with 3, 3′-diaminobenzidine. For the TH-positive neurons counting, four representative areas per well of the 24-well plate were counted. In each treatment group, three wells were used for cell counting [19].

### 2.7. BDNF and GDNF Measurement by ELISA

The BDNF and GDNF levels in the culture medium were quantified with enzyme-linked immunosorbent assay (ELISA) kits according to the procedures provided by the manufacturer.

### 2.8. Statistical Analysis

Results were expressed as mean ± SEM from three independent experiments performed in triplicate. Statistical significance was analyzed by one- or two-way ANOVA using GraphPad Prism software (GraphPad Software Inc., San Diego, CA, USA). When ANOVA indicated the significant differences, pairwise comparisons between means were accessed by Bonferroni's posttest with correction. A value of *P* < 0.05 was considered statistically significant.

## 3. Results

### 3.1. Resveratrol Produced Neurotrophic Effects on Cultured Dopamine Neurons

**Figure 1:**
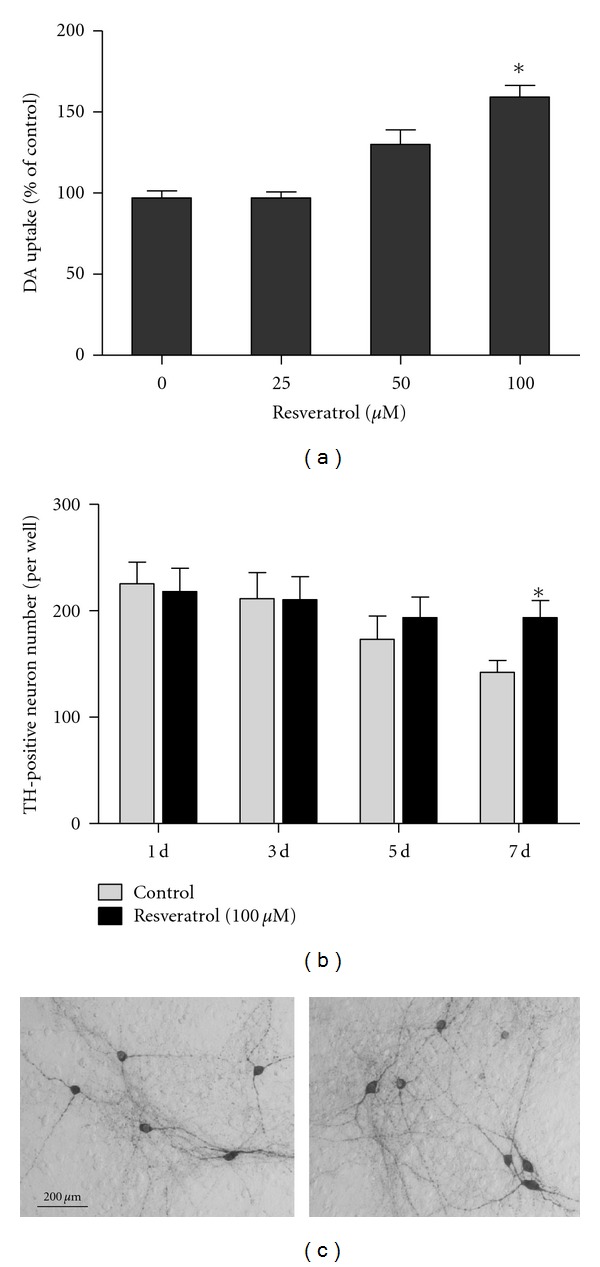
Resveratrol produced neurotrophic effects on cultured DA neurons. Rat primary midbrain neuron-glia cultures were treated with different concentration of resveratrol on the first day. Seven days later, the neurotrophic effects were determined by [^3^H] DA uptake assay (a) and TH-positive neuron counting using the immunocytochemical analysis (b). Representative images of immunostaining 7 days after resveratrol treatment from three experiments were shown (c). Scale bar, 200 *μ*m. Results were expressed as a percentage of the control cultures and were the mean ± SEM from three independent experiments performed in triplicate. *P < 0.05 compared with the control cultures.

Rat primary midbrain neuron-glia cultures were treated with resveratrol (25–100 *μ*M) on the first day. Seven days later, the neurotrophic effects were determined by [^3^H] DA uptake assay and immunocytochemical staining. The [^3^H] DA uptake assay indicated that resveratrol treatment increased the capacity of dopaminergic neurons to take up DA by approximately 160% compared with the control cultures (Figure 1(a)). To investigate the time course of resveratrol-mediated neurotrophic effects, TH-positive neuronal counting was applied 1, 3, 5, and 7 days after resveratrol treatment on the first day. A time-dependent decrease of TH-positive neurons was observed in the control cultures. However, there was no further dopaminergic neuronal loss at days 5 and 7 in resveratrol-treated cultures (Figure 1(b)). From the morphological analysis, resveratrol treatment caused an apparent increase in the neurite and number of DA neurons (Figure 1(c)). 

### 3.2. Astroglia Were Responsible for Resveratrol-Mediated Neurotrophic Effects

**Figure 2:**
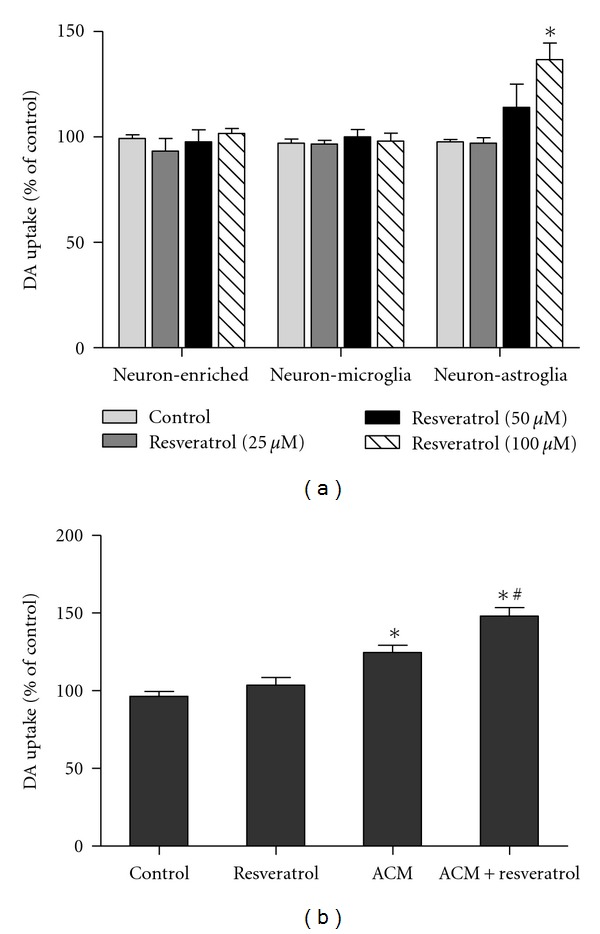
Astroglia were responsible for resveratrol-mediated neurotrophic effects. Three types of cultures including neuron-enriched, neuron-astroglia, and neuron-microglia reconstituted cultures were treated with resveratrol (100 *μ*M) on the first day. The [^3^H] DA uptake assessment was performed 7 days after resveratrol administration (a). The astroglia-conditioned medium (ACM) was prepared from primary astroglia cultures treated with 100 *μ*M resveratrol (ACM-resveratrol) or control (ACM) for 48 h. ACM and ACM-resveratrol were collected and added to the neuron-enriched cultures and incubated for 7 days. The dompaminergic neuronal function was evaluated by the [^3^H] DA uptake assay (b). Results were expressed as a percentage of the control cultures and were the mean ± SEM from three independent experiments performed in triplicate. *P < 0.05 compared with the control cultures; ^#^ *P* < 0.05 compared with the ACM cultures.

To investigate which cell type participated in resveratrol-mediated neurotrophic effects, three types of cultures including neuron-enriched, neuron-astroglia, and neuron-microglia reconstituted cultures were prepared and treated with resveratrol (100 *μ*M) on the first day. Seven days later, as shown in Figure 2(a), in neuron-astroglia cultures, but not in either neuron-enriched or neuron-microglia cultures, resveratrol treatment increased DA uptake capacity by approximately 150% compared with the control cultures.

To further confirm the role of astroglia in resveratrol-mediated neurotrophic effects, the astroglia-conditioned medium (ACM) was prepared from primary astroglia-enriched cultures. Primary astroglia were treated with resveratrol (100 *μ*M) and incubated for 48 h. The conditioned medium in the absence (ACM) or presence of resveratrol (ACM-resveratrol) were collected and dialyzed and then added to the neuron-enriched cultures. Seven days later, the [^3^H] DA uptake assay indicated that ACM and ACM-resveratrol increased DA uptake capacity compared with the control group in the neuron-enriched cultures. Additionally, compared with ACM-treated cultures, a significant increase in DA uptake capacity was discerned in ACM-resveratrol-treated cultures (Figure 2(b)).

### 3.3. Resveratrol Increased BDNF and GDNF Production in the Culture Medium

**Figure 3:**
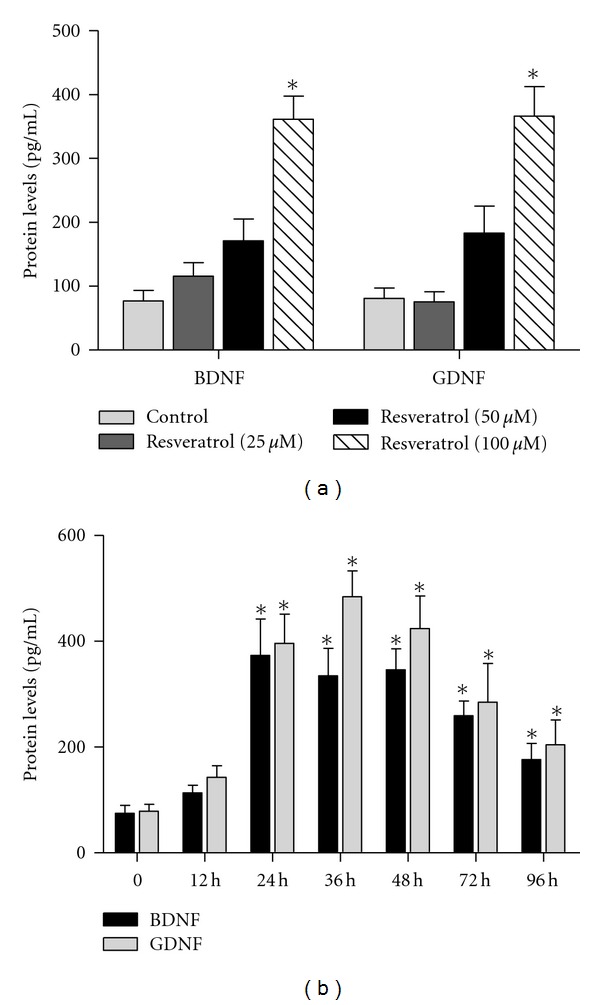
Resveratrol increased BDNF and GDNF production in the culture medium. The production of BDNF and GDNF in the supernatant of primary neuron-glia cultures after resveratrol treatment for 48 h was measured by ELISA (a). Twelve, 24, 36, 48, 72, and 96 h after resveratrol (100 *μ*M) treatment on the first day, the release of BDNF and GDNF in the culture medium was detected by ELISA (b). Results were the mean ± SEM from three independent experiments performed in triplicate. *P < 0.05 compared with the control cultures.

Rat primary neuron-glia cultures were treated with resveratrol (25–100 *μ*M) for 48 h. The BDNF and GDNF production in the culture medium was detected by ELISA. As shown in Figure 3(a), resveratrol induced BDNF and GDNF release in the supernatant compared with the control cultures. 

We next investigated whether resveratrol time-dependently increased BNDF and GDNF production. Twelve, 24, 36, 48, 72, and 96 h after resveratrol treatment on the first day, the BDNF and GDNF release in the culture medium was measured by ELISA. As shown in Figure 3(b), resveratrol induced BDNF production in 12 h, peaked at 24 h and then decreased until 96 h. However, the GDNF release was initially increased at 24 h, and continued to increase in 36 h and then decreased 48 h after resveratrol treatment.

### 3.4. Astroglial BDNF and GDNF Production Was Involved in Resveratrol-Mediated Neurotrophic Effects

**Figure 4:**
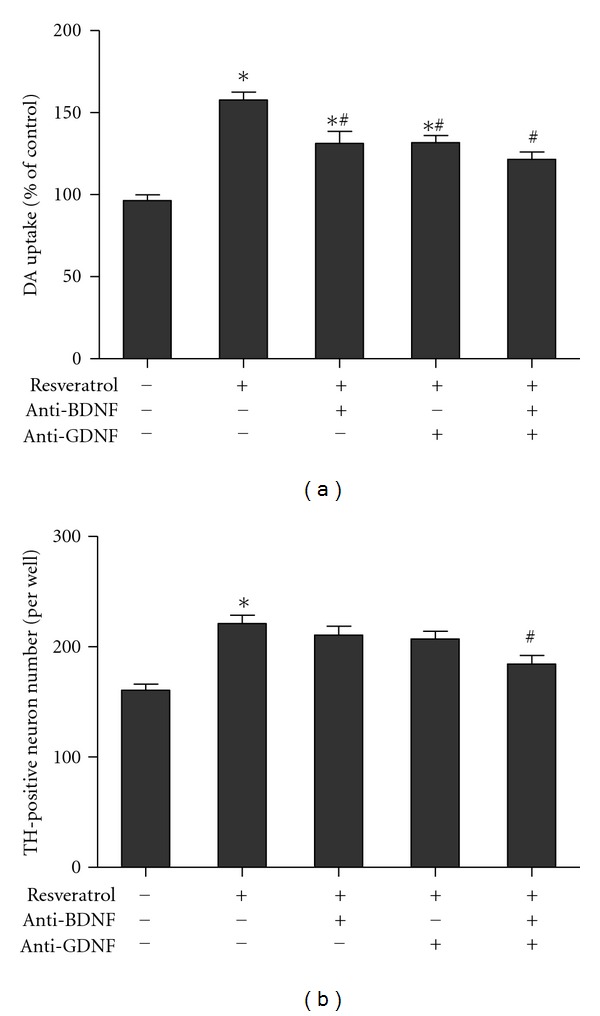
Astroglial BDNF and GDNF production was involved in resveratrol-mediated neurotrophic effects. Primary neuron-glia cultures were pretreated with resveratrol (100 *μ*M) followed by the treatment of anti-BDNF or anti-GDNF antibodies or combined these two antibodies on the first day. Seven days later, the [^3^H] DA uptake assay (a) and TH-positive neuronal counting analysis (b) were performed to determine resveratrol-mediated neurotrophic effects. Results were expressed as a percentage of the control cultures and were the mean ± S.E.M. from three independent experiments performed in triplicate. *P < 0.05 compared with the control cultures, ^#^ *P* < 0.05 compared with resveratrol-treated cultures.

To elucidate the neurotrophic factors such as BDNF and GDNF that were involved in resveratrol-mediated neurotrophic effects, the neutralization assay was applied in the neuron-glia cultures. As shown in Figure 4(a), resveratrol treatment increased DA uptake capacity compared with the control cultures and this increase was inhibited by anti-BDNF, anti-GDNF and anti-BDNF combined with anti-GDNF antibodies. Although there was significant difference between anti-BDNF or anti-GDNF and the control cultures, no significant difference between the control and anti-BDNF combined with anti-GDNF antibody cultures, and between anti-BDNF or anti-GDNF, and anti-BDNF combined with anti-GDNF antibody groups was investigated. Furthermore, TH-positive neuronal counting assay shown in Figure 4(b) indicated that anti-BDNF combined with anti-GDNF antibodies attenuated resveratrol-mediated neuroprotection, whereas single treatment of anti-BDNF or anti-GDNF antibody failed to produce any inhibitory effects. 

## 4. Discussion

The present study showed that resveratrol produced neurotrophic effects on DA neurons in primary midbrain neuron-glia cultures. Furthermore, astroglia-derived neurotrophic factors release was responsible for resveratrol-mediated neurotrophic properties as evidenced by the following observations: (1) resveratrol failed to exert neurotrophic effects on DA neurons in primary cultures without the presence of astroglia; (2) the astroglia-conditioned medium prepared from primary astroglia-enriched cultures treated with resveratrol produced neurotrophic effects in primary neuron-enriched cultures; (3) resveratrol increased neurotrophic factors release in the concentration- and time-dependent manners; (4) resveratrol-mediated neurotrophic effects were suppressed by the inhibition of neurotrophic factors production. These findings suggest that resveratrol could produce neurotrophic effects on cultured DA neurons through prompting astroglial BDNF and GDNF release.

Astroglia, the most abundant cell type in the higher mammalian nervous system, were present in all regions of the brain and appeared to be organized in the strategic positions closely associated with the neuronal structures [20]. A wealth of evidence indicates that the impact of astroglia on the regulation of neurogenesis and synaptogenesis and the modulation of neuronal plasticity has been well documented [21]. Furthermore, upon releasing various neurotrophic factors, astroglia also played the key roles in neuroprotection against neuronal damage and neurodegeneration [22]. Therefore, astroglia were considered to be the important potential target for manipulation [23]. In the present study, resveratrol exerted neurotrophic effects in both primary neuron-glia and neuron-astroglia cultures but not either neuron-enriched or neuron-microglia cultures. Also, the neurotrophic effects were discerned after resveratrol-treated culture medium in astroglia-enriched cultures was added to the neuron-enriched cultures. These findings suggested that astroglia were required for resveratrol-elicited neurotrophic properties. 

Further studies indicated that the increased neurotrophic factors such as BDNF and GDNF production participated in resveratrol-mediated astroglial neurotrophic effects. It has been demonstrated that the neurotrophic factors are responsible for the development, maintenance, and survival of neurons, microglia, astroglia, and oligodendrocytes [4]. A large body of evidence showed that BDNF modulated synaptic plasticity and consequently improved the cognition, learning, and memory formation in several animal models [24]. Studies also indicated that the decreased BDNF level was observed in chronic pain, depression, epilepsy, and neurodegenerative diseases such as Alzheimer's disease, PD, and Huntington's disease [25]. In addition to BDNF, GDNF was found to protect neuronal damage against ischemia, drug abuse, and pain [26]. Based on the animal studies, GDNF had been found to protect DA neurons in 6-OHDA- and MPTP-induced PD models [27, 28]. Moreover, the GDNF protein expression was downregulated in the peripheral blood and postmortem brain tissue of patients with mood disorders [29, 30]. Taken together, promotion of BDNF and GDNF release are recognized to hold a promising therapeutic strategy for the treatment of neurological disorders. The present study revealed that resveratrol concentration- and time-dependently increased BDNF and GDNF release in primary neuron-glia cultures. Similar results shown that resveratrol significantly induced BDNF gene expression in the hippocampus of rat brain and increased BDNF and GDNF production in primary astroglia-enriched cultures [31, 32]. In addition, It is interesting to note that although the application of either anti-BDNF or anti-GDNF antibodies inhibited resveratrol-mediated neurotrophic effects in primary neuron-glia cultures, the partial neurotrophic effects were still presented compared with the control cultures. Furthermore, treatment of anti-BDNF combined with anti-GDNF antibodies could neutralize resveratrol-induced neurotrophic effects. Overall, probably resveratrol-elicited neurotrophic effects were not attributed to a single neurotrophic factor action, and it is very likely that the multiple neurotrophic factors release might account for resveratrol-mediated neurotrophic properties.

Several clinical trials aimed at reviving DA neurons through direct infusion of GDNF to PD patient brains were conducted in the past few years. However, few of these clinical trials succeeded due to the occurrence of severe sided effects [33–36]. In conclusion, the current study supported that astroglia were indispensable for the modulation of the survival and the function of DA neurons and provided a new therapeutic potential targeting astroglia for PD treatment. Instead of direct delivery of a single neurotrophic factor into the brain, resveratrol-mediated neurotrophic factors release and neurotrophic effects might open new alternative avenues for neurotrophic factor-based therapy for PD.

## References

[1] Gao HM, Liu B, Zhang W, Hong JS (2003). Novel anti-inflammatory therapy for Parkinson’s disease. *Trends in Pharmacological Sciences*.

[2] Block ML, Zecca L, Hong JS (2007). Microglia-mediated neurotoxicity: uncovering the molecular mechanisms. *Nature Reviews Neuroscience*.

[3] Ranaivo HR, Craft JM, Hu W (2006). Glia as a therapeutic target: selective suppression of human amyloid-*β*-induced upregulation of brain proinflammatory cytokine production attenuates neurodegeneration. *Journal of Neuroscience*.

[4] Wu HM, Tzeng NS, Qian L (2009). Novel neuroprotective mechanisms of memantine: increase in neurotrophic factor release from astroglia and anti-inflammation by preventing microglial activation. *Neuropsychopharmacology*.

[5] Maragakis NJ, Rothstein JD (2006). Mechanisms of Disease: astrocytes in neurodegenerative disease. *Nature Clinical Practice Neurology*.

[6] Chen PS, Peng GS, Li G (2006). Valproate protects dopaminergic neurons in midbrain neuron/glia cultures by stimulating the release of neurotrophic factors from astrocytes. *Molecular Psychiatry*.

[7] Sullivan AM, Toulouse A (2011). Neurotrophic factors for the treatment of Parkinson’s disease. *Cytokine and Growth Factor Reviews*.

[8] Phillips HS, Hains JM, Armanini M, Laramee GR, Johnson SA, Winslow JW (1991). BDNF mRNA is decreased in the hippocampus of individuals with Alzheimer’s disease. *Neuron*.

[9] Schindowski K, Belarbi K, Buée L (2008). Neurotrophic factors in Alzheimer’s disease: role of axonal transport. *Genes, Brain and Behavior*.

[10] Saiko P, Szakmary A, Jaeger W, Szekeres T (2008). Resveratrol and its analogs: defense against cancer, coronary disease and neurodegenerative maladies or just a fad?. *Mutation Research*.

[11] Zhang F, Liu J, Shi JS (2010). Anti-inflammatory activities of resveratrol in the brain: role of resveratrol in microglial activation. *European Journal of Pharmacology*.

[12] Markus MA, Morris BJ (2008). Resveratrol in prevention and treatment of common clinical conditions of aging. *Clinical Interventions in Aging*.

[13] Moriya J, Chen R, Yamakawa JI, Sasaki K, Ishigaki Y, Takahashi T (2011). Resveratrol improves hippocampal atrophy in chronic fatigue mice by enhancing neurogenesis and inhibiting apoptosis of granular cells. *Biological and Pharmaceutical Bulletin*.

[14] Zhang F, Shi JS, Zhou H, Wilson B, Hong JS, Gao HM (2010). Resveratrol protects dopamine neurons against lipopolysaccharide-induced neurotoxicity through its anti-inflammatory actions. *Molecular Pharmacology*.

[15] Zhang F, Lu YF, Wu Q, Liu J, Shi JS (2012). Resveratrol promotes neurotrophic factor release from astroglia. *Experimental Biology and Medicine*.

[16] Liu B, Du L, Hong JS (2000). Naloxone protects rat dopaminergic neurons against inflammatory damage through inhibition of microglia activation and superoxide generation. *Journal of Pharmacology and Experimental Therapeutics*.

[17] Zhang W, Shin EJ, Wang T (2006). 3-Hydroxymorphinan, a metabolite of dextromethorphan, protects nigrostriatal pathway against MPTP-elicited damage both in vivo and in vitro. *FASEB Journal*.

[18] Gao HM, Hong JS, Zhang W, Liu B (2002). Distinct role for microglia in rotenone-induced degeneration of dopaminergic neurons. *Journal of Neuroscience*.

[19] Zhang F, Qian L, Flood PM, Shi JS, Hong JS, Gao HM (2010). Inhibition of I*κ*B kinase-*β* protects dopamine neurons against lipopolysaccharide-induced neurotoxicity. *Journal of Pharmacology and Experimental Therapeutics*.

[20] Rappold PM, Tieu K (2010). Astrocytes and therapeutics for Parkinson’s disease. *Neurotherapeutics*.

[21] Ullian EM, Christopherson KS, Barres BA (2004). Role for glia in synaptogenesis. *GLIA*.

[22] Darlington CL (2005). Astrocytes as targets for neuroprotective drugs. *Current Opinion in Investigational Drugs*.

[23] Barreto G, White RE, Ouyang Y, Xu L, Giffard RG (2011). Astrocytes: targets for neuroprotection in stroke. *Central Nervous System Agents in Medicinal Chemistry*.

[24] Pardon MC (2010). Role of neurotrophic factors in behavioral processes: implications for the treatment of psychiatric and neurodegenerative disorders. *Vitamins and Hormones*.

[25] Pezet S, Malcangio M (2004). Brain-derived neurotrophic factor as a drug target for CNS disorders. *Expert Opinion on Therapeutic Targets*.

[26] Linker R, Gold R, Luhder F (2009). Function of neurotrophic factors beyond the nervous system: inflammation and autoimmune demyelination. *Critical Reviews in Immunology*.

[27] Tang FI, Tien LT, Zhou FC, Hoffer BJ, Wang Y (1998). Intranigral ventral mesencephalic grafts and nigrostriatal injections of glial cell line-derived neurotrophic factor restore dopamine release in the striatum of 6-hydroxydopamine-lesioned rats. *Experimental Brain Research*.

[28] Cheng FC, Ni DR, Wu MC, Kuo JS, Chia LG (1998). Glial cell line-derived neurotrophic factor protects against 1-methyl- 4-phenyl-1,2,3,6-tetrahydropyridine (MPTP)-induced neurotoxicity in C57BL/6 mice. *Neuroscience Letters*.

[29] Michel TM, Frangou S, Camara S (2008). Altered glial cell line-derived neurotrophic factor (GDNF) concentrations in the brain of patients with depressive disorder: a comparative post-mortem study. *European Psychiatry*.

[30] Takebayashi M, Hisaoka K, Nishida A (2006). Decreased levels of whole blood glial cell line-derived neurotrophic factor (GDNF) in remitted patients with mood disorders. *International Journal of Neuropsychopharmacology*.

[31] Wei R, Lin CM, Tu YY (2010). Strain-specific BDNF expression of rat primary astrocytes. *Journal of Neuroimmunology*.

[32] Rahvar M, Nikseresht M, Shafiee SM (2011). Effect of oral resveratrol on the BDNF gene expression in the hippocampus of the rat brain. *Neurochemical Research*.

[33] Yan M, Dai H, Ding T (2011). Effects of dexmedetomidine on the release of glial cell line-derived neurotrophic factor from rat astrocyte cells. *Neurochemistry International*.

[34] Kordower JH, Palfi S, Chen EY (1999). Clinicopathological findings following intraventricular glial-derived neurotrophic factor treatment in a patient with Parkinson’s disease. *Annals of Neurology*.

[35] Nutt JG, Burchiel KJ, Comella CL (2003). Randomized, double-blind trial of glial cell line-derived neurotrophic factor (GDNF) in PD. *Neurology*.

[36] Peterson AL, Nutt JG (2008). Treatment of Parkinson’s disease with trophic factors. *Journal of the American Society for Experimental NeuroTherapeutics*.

